# Effect of Variable Energy Served on 24-Hour Energy Intake in 16 Preschools, Chicago, Illinois, 2007

**Published:** 2011-04-15

**Authors:** Debbie A. Lown, Marian L. Fitzgibbon, Alan Dyer, Linda Schiffer, Sandra Gomez, Carol L. Braunschweig

**Affiliations:** Department of Biomedical Sciences, Grand Valley State University. Dr Lown is also affiliated with the Department of Human Nutrition, University of Illinois at Chicago, Chicago, Illinois.; Jesse Brown VA Medical Center and University of Illinois at Chicago, Chicago, Illinois; Northwestern University, Chicago, Illinois; University of Illinois at Chicago, Chicago, Illinois; University of Illinois at Chicago, Chicago, Illinois; University of Illinois at Chicago, Chicago, Illinois

## Abstract

**Introduction:**

The effect of varying portion size in a natural environment on the self-regulation of 24-hour energy intake has not been evaluated. We studied students at 16 preschools in Chicago to determine the effect of meals with variable energy and macronutrients on the amount consumed over a 24-hour period (ie, at preschool and outside of preschool).

**Methods:**

The food items and portion sizes served at 16 preschools in the Chicago Public Schools early childhood education programs were observed for 3 to 8 days. Children were assessed for total energy and selected macronutrients consumed at preschool and outside of preschool for a 24-hour period; their data were pooled and reported by school.

**Results:**

The students were predominantly African American (96%); on average, 32 students (range, 21-38) participated at each of the 16 preschools, and the age range of students was 4.0 to 4.5 years. The energy served at preschools ranged from 48% to 90% of the daily recommended energy allowance (REA). The mean energy intake at school was significantly higher (39% of REA) at 2 preschools, including 1 that served 90% of the REA. Mean energy consumption outside of preschool and total 24-hour energy consumption did not differ by preschool, adjusting for body mass index *z* score and sex.

**Conclusion:**

The preschools served meals that widely varied by portion size and energy; however, this variation did not result in differences in mean 24-hour nutrient intakes for the students attending these schools.

## Introduction

The prevalence of obesity in children aged 2 to 5 years increased from 7% in 1988 to 12% in 2006 (1,2). This increase is a public health issue because overweight children tend to become overweight adults (3,4), and adult obesity increases the risk of illness and premature death (5,6).

Since the late 1970s, the average portion size that children consume both in and outside of the home has increased (7). In children older than 6 years, but not children aged 3 to 5 years, meal portion size is positively associated with body mass index (BMI) (8). In single-meal protocols in which the portion sizes of entrees are manipulated in a laboratory setting, children aged 5 years or older and adults increase their meal energy intakes and cumulative daily energy intake with increased portion size (9-11). Increased energy intake in a single meal has also been reported in children younger than 5 years (12,13); however, exposure to larger portion sizes does not increase cumulative daily energy intake in this age group (14). Thus, at least in a laboratory setting, it appears that younger children have highly variable energy intake from meal to meal, but their total daily energy intake remains consistent because of their ability to self-regulate intake (15). No study has examined the effect in a natural environment of varying portion sizes on individual meal or total 24-hour intake in children younger than 5 years.

Because the increasing number of single-parent and dual-income homes, approximately 73% of children younger than 5 spend some time each day in nonparental care (16). Approximately 1 million young children receive their breakfast and lunch from the federal Head Start program. The Head Start program is mandated to follow the Child and Adult Care Food Program's (CACFP's) meal patterns and minimum serving sizes (www.fns.usda.gov/cnd/) to provide approximately 25% and 33% of the Recommended Dietary Allowance for energy at breakfast and lunch, respectively, and no more than 30% of calories from fat (17). Studies examining the nutrient content of menus served to preschool children found that, despite adherence to the meal pattern guidelines, insufficient amounts of total energy and micronutrients and excessive energy as fat (approximately 40% of total energy) were served (18,19).

In this cross-sectional analysis we describe the variation in mean energy and macronutrients served by 16 preschools in Chicago and the effect of this variation on the mean amount of energy and selected macronutrients consumed over a 24-hour period (ie, at preschool and outside of preschool).

## Methods

### Study design and setting

The teacher-delivered Hip-Hop to Health Jr. Obesity Prevention Effectiveness Trial is based on the National Institutes of Health's efficacy trial, Hip-Hop to Health Jr. (20-22). That trial compared a teacher-delivered weight control intervention with a teacher-delivered general health intervention targeting children aged 3 to 5 years enrolled in 18 Head Start programs administered through the Chicago Public Schools. We present the baseline data collected in the fall of 2007 for the 16 schools (n = 539 students) that agreed to participate in the nutrition component of this trial. The participating schools varied in number of students and grade levels taught; however, because all Head Start programs were designed to be separate from the rest of the school, these variations affected the preschool atmosphere minimally. Children were excluded from the study if they had no medical clearance to participate in the program's physical activity component or if they required a special diet. Children were also excluded if dietary intake data (at preschool or outside of preschool; n = 23) were missing. Data were analyzed on 516 participants at these 16 schools. The University of Illinois at Chicago's institutional review board approved the protocol, and written informed consent was obtained from each participant's parent or legal guardian.

### Measures

**Student characteristics**

Parents reported their child's date of birth, sex, and race. If the parent could not be interviewed, the child's teacher reported the date of birth. Sex and race were determined by observation. Research staff measured height by using a Seca 214 portable stadiometer (Seca Corp, Hanover, Maryland). Weight was measured by using a Tanita BWB-800 digital scale (Tanita Corporation of America, Inc, Arlington Heights, Illinois). Children removed their shoes and any heavy outer clothing for the anthropometric measurements. Both height and weight were measured twice to the nearest 0.1 cm and 0.1 kg, respectively. BMI *z* scores and BMI percentiles for age and sex were calculated for each child based on the 2000 Centers for Disease Control and Prevention (CDC) growth charts (23) by using CDC's program for SAS (SAS Institute, Inc, Cary, North Carolina) (24).

**Measurement of nutrient content of served preschool meals**

Research staff with undergraduate degrees in nutrition observed food items served at each meal for 3 to 5 days at all preschools. The nutrient data and preparation techniques for each food item served were available from the Chicago Public Schools to determine energy and macronutrients in the food. The mean daily energy and macronutrients served were calculated for each preschool.

**Measurement of participants' dietary intake**

**Intake at preschool.** Research staff unobtrusively documented the amount of food both served and consumed for all meals for 3 to 5 children per classroom each day for 3 to 8 days. The food and beverages consumed by each child were determined by calculating the difference between the amount of food served and the amount remaining on their tray at the end of each meal. All remaining food on the trays was measured with measuring spoons and liquid measuring cups as appropriate. The mean nutrient intake at each preschool was calculated from the children's average amount of energy and micronutrients consumed.

**Intake outside of preschool.** A food record recall method was used to obtain diet intake outside of school. The day before a child was to be observed, a packet was provided to the child's parent or guardian containing a food record form for recording all food consumed outside of preschool and a food portion visual guide to assist in estimating the amount of foods eaten. Research staff instructed the parents on completion of the food records and asked parents to record all food and drinks consumed by their child outside of school (ie, that afternoon and evening and the following morning before school). Each record was reviewed by the research staff for completeness and clarification by using the 5-step multiple-pass method (25) on the morning of the child's classroom observation. To maximize participation, parents who did not return food record forms before their child's scheduled observation were contacted by telephone and the 5-step multiple-pass method was used to determine the foods consumed by the child during the previous evening and before coming to school that day. The mean outside-of-preschool intake was calculated from the children's average energy and micronutrient intake at each school.

**24-Hour intake.** Total daily intake for each school was calculated by adding food intake inside and outside of preschool over a 24-hour interval; intake was assessed only on school days. Each child contributed one 24-hour intake. The mean energy intake for each preschool was compared with the daily recommended energy allowance (REA) based on the group's average age and assuming an *active* physical activity level (26). In this group of children, the estimated daily REA was 1,656 calories (90 kcal/kg). The dietary intake data were processed with Nutrition Data System for Research (University of Minnesota Nutrition Coordinating Center, Minneapolis, Minnesota).

### Statistical analyses

Data analyses were performed with SAS version 9.1. The primary outcomes of interest were average energy and macronutrients 1) served and consumed at preschool, 2) consumed outside of preschool, and 3) consumed during the total 24-hour period, by preschool program. Dietary data were pooled and analyzed at the preschool level because energy and macronutrients served were observed by preschool, not individual children. Descriptive statistics including the mean, standard deviation, and range were generated for the 16 preschool programs. Because the variability in energy and macronutrient intake between preschools may be due to characteristics of the group of children, *χ* ^2^ tests were used to assess differences between the preschool programs by sex. Spearman correlation coefficients were used to test for associations between BMI and BMI *z* score and other variables. Mean age and mean BMI *z* scores of the groups of children at each preschool were compared by using the Wilcoxon rank sum test. Mean nutrient differences between preschools for 1) nutrients served, 2) at-preschool intake, 3) outside-of-preschool intake, and 4) total 24-hour intake by preschool program were determined by using analysis of covariance with mean BMI *z* score and sex as covariates. This adjustment was made to control for differences in preschools because the ability to regulate intake has been associated with weight in preschool children (27). BMI and age as covariates were also explored; however, the data are reported with sex and mean BMI *z* score, because results did not differ. Tukey's honestly significant difference (HSD) test was used for pairwise comparisons of means at the preschool level. Mean energy served at each preschool, energy intake at preschool, energy intake outside of preschool, and total 24-hour energy intake at the 16 preschool programs were compared with the average daily REAs based on mean age and weight-specific estimated energy requirements for the group (26). Data are presented as preschool group mean and standard deviation with statistical significance set at *P* < .05 for differences between the preschools.

## Results

Most of these preschool students were African American (96%), and all had incomes that qualified for participation in Head Start programs. No significant difference was observed between preschools for mean BMI *z* score (Table 1), age (range, 4.0-4.5 y), or sex. Preschool calories were correlated with BMI and BMI *z* score; however, total calories and age were not. Boys had a significantly higher mean school calorie intake than girls.

### Mean nutrient content of meals served at preschool

Approximately 11 eating events (breakfasts, lunches, and snacks) were observed for each school. The mean energy served and percentage of REA ranged from 799 to 1,494 kcal and 48% to 90% of the REA (Table 2). Three preschools served more than the recommended 67% of REA for energy, and 4 preschools served less than 55% of the REA. One preschool (preschool 1) served significantly more mean energy than 3 other preschool sites (for preschools 2 and 7, *P* = .02, and for preschool 8, *P* = .01, Tukey's HSD test for pairwise comparison). Preschool 1 also served meals consisting of significantly higher mean grams of carbohydrate than all other schools. More than half of the schools exceeded the recommended calories from fat (<30%) (26); the range was 25% to 40% of calories. There was no significant difference in the mean grams of protein and fiber served by each school, and all the schools served more mean protein and less mean fiber than recommended (26).

### Mean nutrient intake at preschool

On average, 32 students were observed at each preschool. The children were not required to consume any meal; thus the number of observed children for each eating event varies and reflects the number of children who consumed each meal or snack (Table 3).

The energy consumed at preschool averaged 33% (range, 25%-39%) of the REA (1,1,656 kcal/d) (Table 4). The high amount of energy (mean [SD] = 1,494 [82] kcal) served at preschool 1 resulted in significantly higher mean energy consumption (39% of REA). Children at this school consumed significantly greater energy (mean [SD] = 647 [221] kcal) while at school compared with children at 2 other schools (*P* = .001 for preschool 11 and *P* = .01 for preschool 12), where a lower amount of energy was served, adjusting for BMI *z* score and sex. The mean percentage of calories from fat consumed at each school ranged from 21% to 40% of calories; children at 11 of the schools consumed more than 30% of calories from fat.

### Mean intake outside of preschool

The mean outside-of-preschool intake was 58% (range, 53%-76%) of the REA. This did not differ by preschool, adjusting for BMI *z* score and sex. The percentage of total calories from fat consumed outside of the preschool was more than 30% (mean, 35%; range, 31%-42%) for all of the preschools.

### 24-Hour mean intake

Despite the highly variable mean energy intake at school, the mean 24-hour energy intake did not differ by preschool, adjusting for BMI *z* score and sex (Table 4). Daily energy consumed was 95% (range, 85%-105%) of the average REA. The percentage of calories consumed as fat ranged from 26% to 37%, and 14 of the 16 of preschools had more than 30% of total daily mean calories consumed from fat. The mean 24-hour protein intake exceeded recommendations (range, 53-71 g), and mean fiber intake (range, 9-12 g) was below the adequate intake recommendation of 19 g (26).

## Discussion

This study contributes to the research on nutritional quality in Head Start programs and is the first to report the effect of highly variable portion sizes on immediate and 24-hour energy intake in groups of preschool children who are in their natural environment. Our findings suggest that serving energy to a group of preschool children at excessive levels (90% of daily needs served at 1 school) may contribute to loss of immediate self-regulation of intake (while at school) but not total 24-hour energy intake. Previous laboratory studies manipulated the size of a single entree (macaroni and cheese) from reference portion size to double portion size and reported an increase in energy meal intake in children aged 2 to 9 years (12-14). Fisher et al tested the effect of doubling the portion size of certain foods on energy intakes over a 24-hour period in low-income 5-year-old Hispanic and African American children in a clinical research setting (10). They found 24-hour energy intake increased 12% in the large-portion condition compared with the reference condition. Since our observations were at the preschool level rather than the individual level, comparison of results from previous investigations is limited. Our study suggests that future research on the effect of large portion sizes on calorie intake in young children should occur in the children's natural environments, where peer and parent-child interactions might result in adjustment of 24-hour energy intake.

Prior studies of Head Start programs have found inadequate provision of energy, vitamins, and minerals and excess calories from fat (18,28,29). Our study confirms the higher-than-recommended calories from fat. The number of preschools providing excessive fat is concerning given the role of fat intake in the development of obesity (30,31) and its potential for displacing intake of food more dense in micronutrients (32).

Our study has several limitations. Data on calorie intake while outside of school relied on reporting from the children's parents. Research has documented underreporting of energy intake by as much as 37% in parents of African American children (33). Efforts made to minimize underreporting included the use of food records and follow-up review of the intakes recorded by parents. It is unlikely underreporting affected our primary finding that the children self-regulated their school calorie intake despite highly variable portions and energy provided. Another limitation of this study was the use of estimated energy requirement for school sites based on reference age, weights for study participants, and *active* level of physical activity, rather than calculated energy needs with actual activity levels for each child. Our estimates of energy requirements were in line with the reported total mean (SD) energy expenditure of 1,379 (290) kcal per day by double-water labeling in 4- to 6-year-olds (34). Furthermore, our energy outcomes were reported for groups rather than individuals; thus, we do not believe this limitation to be a major concern. Finally, our analysis is cross-sectional, based on the intake on a single 24-hour school day to estimate the usual intake for these groups of preschool children; therefore, we are unable to state the long-term causal effect of preschool meals on usual nutrient intake.

Our study indicates that, at the group level, a consistent mean 24-hour energy intake occurred through self-regulation of intake at school and outside of school despite variable levels of energy served in an urban early childhood education program. Our data suggest the need for education and training of Head Start staff on providing appropriate serving sizes for children, reducing the fat content of meals, and allowing self-selected serving sizes of nutritious foods that meet the Dietary Guidelines for Americans.

## Figures and Tables

**Table 1:** Mean BMI *z* Scores and Percentage of Preschool Students With a BMI at the 85th Percentile or Higher, by Preschool, Chicago, Illinois, 2007

**Preschool**	Mean BMI *z* Score^a^ (SD)	BMI ≥85th percentile, %
1 (n = 38)	0.95 (0.9)	47
2 (n = 38)	0.50 (0.9)	29
3 (n = 31)	0.40 (1.2)	23
4 (n = 38)	0.70 (0.9)	39
5 (n = 29)	0.74 (1.2)	34
6 (n = 34)	0.42 (1.1)	26
7 (n = 29)	0.75 (1.1)	41
8 (n = 32)	0.70 (1.2)	28
9 (n = 28)	0.71 (0.9)	43
10 (n = 36)	0.17 (0.8)	11
11 (n = 21)	0.87 (1.4)	33
12 (n = 38)	0.76 (1.0)	31
13 (n = 33)	0.66 (0.8)	36
14 (n = 34)	0.50 (1.0)	32
15 (n = 29)	0.46 (1.1)	34
16 (n = 28)	0.54 (1.0)	25

Abbreviations: BMI, body mass index; SD, standard deviation

a Unadjusted mean

**Table 2:** Mean Energy, Selected Macronutrients, and Percentage of REA Served by Preschools During a 3- to 5-Day Period, Chicago, Illinois, 2007

**Pre-school**	Mean Energy Served,^a^ kcal (SD)	% REA^b^	Mean Protein Served,^a^ g (SD)	% Energy as Protein	Mean Carbohydrate Served,^a^ g (SD)	% Energy as Carbohydrate	Mean Fat Served,^a^ g (SD)	% Energy as Fat	Mean Fiber Served,^a^ g (SD)
1	1,494 (82)	90	55 (13)	15	226 (23)	61	44 (9)	27	14 (2)
2	867 (94)^c^	52	37 (4)	17	122 (16)^d^	56	28 (6)	29	9 (2)
3	956 (40)	58	44 (19)	18	124 (53)^d^	52	33 (16)	31	8 (4)
4	1,074 (116)	65	48 (3)	18	149 (11)^d^	55	34 (6)	28	7 (2)
5	1,022 (101)	62	50 (11)	20	122 (22)^d^	48	39 (6)	34	9 (1)
6	896 (182)	54	33 (11)	15	132 (16)^d^	59	27 (9)	27	5 (3)
7	825 (180)^c^	50	39 (18)	19	107 (24)^d^	52	27 (11)	29	7 (6)
8	799 (110)^c^	48	36 (16)	18	107 (9)^d^	54	27 (11)	30	7 (3)
9	977 (107)	59	49 (10)	20	135 (10)^d^	55	28 (4)	26	8 (1)
10	1,061 (134)	64	56 (3)	21	125 (24)^d^	47	39 (10)	33	8 (3)
11	980 (324)	59	44 (24)	18	117 (11)^d^	48	39 (23)	36	8 (3)
12	1,274 (171)	77	54 (15)	17	139 (10)^d^	44	57 (11)	40	10 (2)
13	1,051 (189)	63	36 (12)	14	155 (34)^d^	59	35 (11)	30	9 (6)
14	1,037 (116)	63	38 (7)	15	124 (23)^d^	48	45 (14)	40	8 (3)
15	1,050 (138)	63	43 (10)	16	159 (8)^d^	61	29 (13)	25	9 (4)
16	1,167 (437)	70	56 (28)	19	134 (38)^d^	46	46 (24)	35	7 (3)

Abbreviations: REA, recommended energy allowance; SD, standard deviation; HSD, honestly significant difference.

a Unadjusted mean.

b Calculated as mean energy served at each preschool compared with an estimated daily energy requirement of 1,656 kcal.

c Significantly different from preschool 1 for mean energy served, by Tukey's HSD test for pairwise comparisons; *P* = .01 for preschool 8 and *P* = .02 for preschools 2 and 7.

d Significantly different from preschool 1 for mean carbohydrate served, by Tukey's HSD test for pairwise comparisons; *P* = .002 for preschool 6, *P* = .004 for preschool 9, *P* = .02 for preschool 13, *P* = .03 for preschools 4 and 15, and *P* < .001 for all other preschools.

**Table 3:** Number of Observation Days and Meals Observed at 16 Preschools, Chicago, Illinois, 2007

**Preschool**	No. of Intake Observation Days	No. of Breakfasts	No. of Lunches	No. of Snacks
1	4	37	38	37
2	5	36	37	1
3	5	29	31	29
4	3	35	36	30
5	4	29	29	17
6	3	34	34	26
7	4	26	28	23
8	4	32	32	12
9	3	26	28	21
10	4	34	36	25
11	8	20	20	1
12	6	35	37	0
13	8	3	32	30
14	8	32	34	21
15	5	25	29	24
16	5	23	27	17
Total meals observed		456	508	314

**Table 4:** Mean Energy Intake at School, 24-Hour Energy Intake, and Percentage of Recommended Energy Allowance, by Preschool, Chicago, Illinois, 2007

**Preschool**	Energy Intake at School,^a^ kcal (SD)	% REA^b^	24-Hour Energy Intake,^a^ kcal (SD)	% REA^b^
1	647 (221)	39	1,526 (422)	92
2	474 (188)^c^	29	1,417 (326)	86
3	642 (240)	39	1,638 (545)	99
4	593 (217)^d^	36	1,624 (538)	98
5	587 (192)	35	1,724 (651)	104
6	587 (165)^d^	35	1,542 (404)	93
7	497 (191)^c^	30	1,536 (504)	93
8	530 (186)	32	1,556 (454)	94
9	475 (156)	29	1,562 (411)	94
10	558 (181)	34	1,490 (483)	90
11	408 (165)^c^,^e^	25	1,667 (821)	101
12	473 (250)^c^,^e^	29	1,736 (522)	105
13	471 (176)^c^	28	1,404 (381)	85
14	556 (252)	34	1,709 (733)	103
15	512 (174)	31	1,568 (495)	95
16	603 (284)^d^	36	1,579 (598)	95

Abbreviations: SD, standard deviation; REA, recommended energy allowance; HSD, honestly significant difference.

a Unadjusted mean.

b Calculated as mean energy intake at each preschool compared with an estimated daily energy requirement of 1,656 kcal.

c Significantly lower mean energy intake compared with preschool 3, by Tukey's HSD test for pairwise comparisons; *P* < .001 for preschool 11, *P* = .003 for preschool 12, *P* = .03 for preschool 2, and *P* = .04 for preschools 7 and 13.

d Significantly higher mean energy intake compared with preschool 11, by Tukey's HSD test for pairwise comparisons; *P* = .006 for preschool 16, *P* = .02 for preschool 6, and *P* = .03 for preschool 4.

e Significantly lower mean energy intake compared with preschool 1, by Tukey's HSD test for pairwise comparisons; *P* = .001 for preschool 11 and *P* = .01 for preschool 12.
